# Mortality among very low birth weight infants after hospital discharge in a low resource setting

**DOI:** 10.1186/s12887-018-1226-4

**Published:** 2018-07-21

**Authors:** Yaser Abdallah, Flavia Namiiro, Jolly Nankunda, Jamiru Mugalu, Yvonne Vaucher

**Affiliations:** 1 Department of Paediatrics and Child Health, Makerere University College of Health Sciences, P.O Box 7072, Kampala, Uganda; 2 Department of Paediatrics and Child Health, Mulago National Referral hospital, Kampala, Uganda; 3 Department of Pediatrics, Division of Neonatal/Perinatal Medicine, School of Medicine, University of California, San Diego, USA

**Keywords:** Preterm neonates, Very low birth weight, Mortality

## Abstract

**Background:**

Early discharge of very low birth weight infant (VLBW) in low resource settings is inevitable but to minimize mortality of these infants after discharge we need to identify the death attributes.

**Method:**

A prospective cohort was conducted among 190 VLBW infants discharged from Mulago Special Care Baby Unit (SCBU) with discharge weight of < 1500 g over an 8 months period. These infants were followed up with the aims of determining the proportion dead 3 months after discharge, identifying factors associated and possible causes of death.

Relevant data were captured, transferred in to STATA and imported to SPSS 12.0.1 for analysis. To determine factors associated with mortality bi-variable and multivariable regressions were conducted. A *p*-value of < 0.05 was considered significant and 95% confidence interval was used.

**Results:**

Of the enrolled infants 164 (86.3%) completed follow up. The median gestational age of study participants was 32 weeks (range 26-35 weeks), the mean discharge weight was 1119 g (range 760-1470 g), and 59.8% were small for gestational age (SGA). During follow up 32 (19.5%) infants died. Infants discharged with weight of < 1200 g accounted for 81.2% of the deaths. Majority of the deaths (68.7%) occurred in the first month after discharge. Factors independently associated with mortality were discharge weight < 1000 g (OR 3.10, p 0.015) and not being SGA (OR 3.54, p 0.019). The main causes of death were presumed sepsis 50.0% and suspected cot death (25.0%).

**Conclusion:**

Mortality after hospital discharge among VLBW infants is high. Discharge at weight < 1200 g may not be a safe practice. Measures to prevent sepsis and suspected cot death should be addressed prior to considering early discharge of these infants.

## Background

It is estimated that more than 22 million babies born worldwide annually are of low birth weight (< 2500 g) of whom 95.6% are in the low resource countries and this trend has not changed in the last 10 years [1, 2]. Low birth weight babies account for 60–80% of all neonatal deaths [3, 4]. Preterm VLBW babies’ are at higher odds of mortality even beyond the neonatal period [4, 5]. The prevalence of preterm/low birth weight babies in Uganda is estimated at 10–20% [6]. In Mulago Special Care Baby Unit (SCBU) (National referral hospital of Uganda), VLBW and extremely low birth weight babies account for 13.8% of admission and approximately 50% of these babies die prior to discharge [7].

Very low birth weight babies are usually hospitalized for longer periods for various reasons including respiratory distress, sepsis, feeding intolerance among others [8, 9]. These babies present a challenge to their parents as well as the health care system [9]. Although Kangaroo Mother Care (KMC) has enabled early discharge of low birth weight babies [10], the higher odds of mortality among such babies stretching beyond the neonatal period has made the timing of their hospital discharge a subject of continued debate.

The World Health Organization (WHO) has developed a more objective and flexible discharge criteria for low birth weight babies [11] but these have been difficult to adhere to especially in the developing countries due to limitation in resources. Many resource limited countries lack KMC facilities; in Uganda only 1/10 of health facilities do have facilities for KMC [12] and hence VLBW babies tend to be discharged at much smaller weights. Varying rates of survival of VLBW babies discharged early to continue home KMC have been described [13–16]; the outcome of such babies discharged from Mulago SCBU is not known.

The practice in SCBU is to discharge babies irrespective of their weight but once stable (i.e. off oxygen and antibiotics), gaining weight, having stable body temperature while in Kangaroo care, and tolerating 80 ml/kg/day of feeds (by cup/tube). These babies are followed up on a weekly basis till they attain a weight of 2500 g. Whereas this method of discharge enables reduction in health care system constraint its safety needs to be elaborated. This study was aimed at throwing more light on proportion of VLBW infants discharged from Mulago SCBU with discharge weight of < 1500 g that die, factors associated and possible causes of death.

## Methods

### Setting

This study was conducted at the Mulago Hospital SCBU. Mulago hospital is a National Referral Hospital for Uganda and teaching hospital for Makerere University. Mulago Hospital SCBU is a level II neonatal unit. The unit admits on average 4600 babies annually with approximately 20% being VLBW babies.

The Mulago SCBU has a preterm, term and Kangaroo mother care sections (KMC). The preterm section has 19 incubators and 2 radiant warmers with minimal space between these equipment’s. The KMC section can handle only 4 mother/infant pair at any one time. Due to the large admission volumes sharing of incubators/radiant warmers by babies is the norm.

With respect to staffing, the unit is served by 3 paediatricians, 16 nurses (working in 3 shifts per day), 2 intern and 2–4 Paediatrics resident doctors on rotational basis.

The most VLBW babies are offered during their stay in the unit include intravenous antibiotics and fluids mainly as boluses, phototherapy and nasal gastric tube feeds when needed. Maximum respiratory support available is with fixed expiratory valve Continuous Positive Airway Pressure (CPAP). Vital signs monitoring is done intermittently. Mothers/caregivers are responsible for feeding their babies on a two hourly basis.

Preterm neonates are discharged from the unit once they are off oxygen, tolerating 80mls/kg/day of feeds and have started gaining weight. Due to lack of adequate space in the unit most of the time infants are discharged before regaining birth weight. Preterm neonates discharged are followed up in the preterm clinic which runs twice weekly at the SCBU.

These preterm infants are followed up on weekly basis in the preterm clinic where growth is monitored, supplements (multivitamin and iron) are refilled, feeds are readjusted (maximum feeding volume is 220 ml/kg/day), nasogastric tubes are replaced and unwell infants are readmitted and managed.

### Study population

All infants discharged from Mulago SCBU whose birth weight was ≤1500 g and discharge weight < 1500 g if the parent consented and they had 2 reliable phone contacts.

### Sample size

Sample size was estimated using Open Epi for frequency in population and assuming expected mortality among infants within 3 months of discharge (25.7%) [14].

Sample Size (n) for 95% Confidence Level was 173. Assuming a 10% loss to follow up the final sample size was (n) = 173 + 173*10% = **190.**

### Procedure

All infants of birth weight ≤ 1500 g admitted to Mulago Hospital SCBU between June 2013 and January 2014 were identified by the research assistant within 24 h of birth and gestational age ascertained using the New Ballard Score [17]. For infants who met inclusion criteria and where ready for discharge, the research assistant discussed with parents in detail and obtained written consent. Recruitment was conducted daily till the desired sample size was reached.

Recruited infants were given a study number for identification. A log book was put in place to ease tracking follow up dates for study participants. Phone contacts were exchanged and checked if they were working. Phone contact for principal investigator and research assistant were given to parents to call in case of any concern and parent’s contacts were taken to easy follow up in case infant is not returned to clinic visits for follow up.

Data was captured at discharge and on the weekly follow up clinic by a trained research assistant. Parents who did not return the infants to the follow up clinic were called on phone and advised to return in the next visit. Parents who missed two consecutive visits and those not contactable were considered as lost to follow up.

In the case of parents who did not return to the follow up clinic because the infant had died, details related to death were obtained by research assistant from the parents.

### Data management and analysis

Information captured in the data sheet was checked by the principle investigator for completeness and accuracy. The data sheets were stored in a locker.

Data from the data sheets were entered into the computer using Microsoft Excel program. It was subsequently exported into STATA software package for analysis. Baseline characteristic of study participants were summarized in a table. The survival was computed as a ratio of study participants alive at 3 months after hospital discharge to the total number of study participants who completed study. Univariate analysis was conducted for continuous variables.

*P* values of < 0.05 were considered significant and confidence interval of 95% was used. Results were summarized in tables.

## Results

Over an 8 month period (June 2013 to January 2014) 412 VLBW infants were admitted to the Special Care Baby Unit of whom 164 (39.8%) died. Of those who survived to discharge 190 were recruited; 164 (86%) infants completed study. Infants excluded from the study included 25 with incomplete data, 12 whose parents declined to consent and 21 whose parents had no reliable phone contacts.

Of the infants who were, enrolled and completed the study, the median gestational age was 32 weeks and the mean discharge weight was 1119 g (range 760-1470 g).

Twenty six (26) infants were loss to follow up (Table 1), twelve never returned for review and parents could not be reached on contacts they had left behind. Fourteen returned but by 3 months after discharge 4 had relocated, while 10 could not be reached.

**Table 1:** Baseline characteristics of study participants

Characteristics	Completed follow up *N* = 164(%)	Loss to follow up *N* = 26(%)	*p*-value
Sex			
Male	71 (43.3)	12 (46.1)	
Female	93 (56.7)	14 (53.9)	
Mode of delivery			
SVD	129 (78.7)	19 (73.1)	0.52
Caesarian section	35 (21.3)	7 (26.9)	
Birth Weight			
< 1000 g	12 (7.3)	1 (3.8)	0.52
1000 -1200 g	74 (45.1)	10 (38.5)	0.52
> 1200 g	78 (47.6)	15 (57.7)	
HIV exposure			
Yes	30 (18.3)	4 (15.4)	0.72
No	134 (81.7)	22 (84.6)	
Small for Gestational Age (SGA)			
Yes	98 (59.8)	7 (27.0)	
No	66 (40.2)	19 (73.0)	
Discharge Weight			
< 1000 g	39 (23.8)	6 (23.0)	0.94
1000 -1200 g	77 (47.0)	11 (42.3)	
> 1200 g	48 (29.2)	9 (34.7)	
Duration in continuous kangaroo			
< 1 week	129 (78.7)	24 (92.3)	0.12
1-2 weeks	30 (18.3)	2 (7.7)	
> 2 weeks	5 (3.0)	0 (0.0)	
Maternal age			
< 20 years	23 (14.0)	11 (42.3)	0.001
≥ 20 years	141 (86.0)	15 (57.7)	
Maternal Parity			
Primiparous	52 (31.7)	9 (34.6)	0.76
2–4	89 (54.3)	16 (61.5)	
> 4	23 (14.0)	1 (3.9)	
Maternal education			
None	7 (4.3)	1 (3.8)	0.92
Primary	49 (29.9)	13 (50.0)	0.046
Secondary	81 (49.4)	9 (34.6)	0.16
Tertiary	27 (16.5)	3 (11.5)	0.52
Maternal monthly income			
Unemployed/Nil	77 (47.0)	16 (61.5)	
< 100,000 (Uganda shillings)	84 (51.2)	4 (15.4)	
≥ 100,000 (Uganda shillings)	3 (1.8)	6 (23.1)	0.0002
Parents living together			
Yes	138 (84.1)	18 (69.2)	
No	26 (15.9)	8 (30.7)	0.07
KMC Supported			
yes	34 (79.3)	4 (15.4)	
No	130 (20.7)	10 (38.5)	0.49
Unknown	0 (0.0)	12 (46.1)	
Postmenstrual age at discharge			
CGA < 35 weeks	95 (57.9)	19 (73.0)	0.14
CGA ≥35 weeks	69 (42.1)	7 (27.0)	

There was high loss to follow up among mothers < 20 years of age (11/34 p 0.001) (Table 1) which might be attributed to poor social support and also among mothers who had better income (6/9 *p* 0.0002) which might be attributed to seeking health care somewhere else.

The average duration of hospital stay among study participants was 12.2 days (range 4-42 days). All infants were discharged on multivitamin drops, iron supplementation and oral aminophylline.

A majority of study infants were discharged on breast feeding with topping off through nasogastric tube, 11 were fully breast feeding and 23 fully tube feeding.

Two study infants were on formula feeds, both died, one from anemia and the other from presumed sepsis. Two of the enrolled infants had anomalies; one had a cleft lip and palate and the other had a patent ductus ateriosus with pulmonary stenosis; both died on follow up.

During follow up 32 infants died (Table 2). Likely cause of death was established by asking the mother to explain circumstances at the time of death.

**Table 2:** Mortality and causes of mortality after hospital discharge

Discharge weight (no)	< 1 month	1-2 months	> 2 months	Total (%)
Duration spent at home at time of death				
< 1000 g (35)	9	2	2	13 (37.1%)
1000-1200 g (75)	8	4	2	14 (18.7%)
> 1200 g (49)	5	–	–	5 (10.2%)
TOTAL	22	6	4	32
Causes of death				
Presumed Sepsis	9	4	3	50%
No details	3	1	–	12.5%
Suspected Cot death	8	–	–	25.0%
Congested cardiac failure	1	–	–	3.1%
Suspected Aspiration	1	1	1	9.4%

Sixteen of study participants died from presumed sepsis based on mother’s description of signs suggesting baby was unwell. Ten of these infants were re-hospitalized but died in hospital, 2 died on the way to hospital and 4 had signs of being unwell but parents did not know what to do so infants died at home. Nine infants who died from presumed sepsis died within 1 month of discharge (Table 2).

With regards to eight infants who died from suspected cot death, the mothers described baby being completely well but found dead in the bed; all infants who died from suspected cot death had discharge weight < 1200 g, with four of them at < 1000 g. Four of these infants died at corrected gestational age of < 35 weeks (Table 2). Eight infants who died from suspected cot death died within 1 month of discharge.

Cause of death of 4 infants could not be established. We were only able to ascertain that the infant had died but could not get details from the mother because we could not talk to her in person on phone for one reason or another. The infants who died from suspected aspiration included one who had cleft lip and palate, one extremely low birth weight 760 g whose feeding tube had come out and one who was receiving breast feeding with top up of 40mls 2 hourly.

Discharge weight of < 1000 g, postmenstrual age < 35 weeks and not being small for gestational age were associated with mortality p 0.008, p 0.012 and p 0.001respectively (Table 3) but in the multivariate analysis (Table 4) discharge at postmenstrual age < 35 weeks was not significant.

**Table 3:** Association between baseline characteristics and outcome

Characteristics	Total 164 (%)	Died *N* = 32(%)	Survived 132(%)	*p*-Value
Maternal age				
< 20 years	23 (14.0)	4 (17.4)	19 (82.6)	
≥ 20 years	141 (86.0)	28 (20.0)	113 (80.0)	0.78
Maternal education				
None	7 (4.3)	2 (28.6)	5 (71.4)	0.54
Primary	49 (29.9)	6 (12.2)	43 (87.2)	
Secondary	81 (49.4)	20 (24.7)	61 (75.3)	0.10
Tertiary	27 (16.5)	4 (14.8)	23 (85.2)	
Maternal income/month				
Unemployed/nil	77 (47.0)	14 (18.2)	63 (81.8)	
30-100 K	84 (51.2)	3 (10.0)	27 (90.0)	
> 100 k	3 (1.8)	15 (26.3)	42 (73.7)	0.11
Maternal parity				
Primiparous	52 (31.7)	10 (19.2)	42 (80.8)	
2–4	89 (54.3)	17 (19.1)	72 (80.9)	
> 4	23 (14.0)	5 (21.7)	18 (78.3)	0.77
Sex				
Male	71 (43.3)	18 (25.3)	53 (74.7)	0.10
Female	93 (56.7)	14 (15.0)	79 (85.0)	
Discharge weight				
< 1000 g	37 (22.6)	13 (34.1)	24 (64.9)	0.008
1000-1199 g	73 (44.5)	13 (17.8)	60 (82.2)	
≥ 1200 g	54 (32.9)	6 (11.1)	48 (88.9)	
Postmenstrual age at discharge				
< 35 weeks	95 (57.9)	25 (26.3)	70 (73.7)	0.012
≥ 35 weeks	69 (42.1)	7 (10.1)	62 (89.9)	
Period in KMC				
< 1 Week	130 (79.3)	20 (15.4)	110 (84.6)	
1-2 weeks	29 (17.7)	9 (31.0)	20 (69.0)	0.09
> 2 weeks	5 (3.0)	3 (60.0)	2 (40.0)	0.04
KMC support				
Supported	34 (79.3)	5 (15.6)	29 (84.4)	
Not supported	130 (20.7)	27 (20.7)	103 (79.3)	0.43
HIV exposure				
Yes	30 (18.3)	7 (23.3)	23 (76.6)	0.56
No	134 (81.7)	25 (18.6)	109 (81.3)	
Parents living together				
Yes	139 (84.8)	24 (17.3)	115 (82.7)	
No	25 (15.2)	8 (32.0)	17 (68.0)	0.09
SGA				
Yes	98 (59.8)	11 (11.2)	87 (88.8)	
No	66 (40.2)	21 (31.8)	45 (68.2)	0.001

**Table 4:** Multivariable analysis on factors independently associated with mortality

Variable	OR	Std. Err.	(95% CI)	*P*
Discharge weight < 1000 g	3.10	1.44	1.24–7.74	0.015
Not SGA	3.54	1.90	1.23–10.1	0.019
Discharge postmenstrual age < 35	1.13	0.68	0.34–3.70	0.838

Although there was a trend towards parents living together improving chances of survival (139/163) it was not statistically significant (Table 3). None of the other social demographic characteristics was found to be associated with outcome.

## Discussion

From this study we observed a mortality rate of 19.5% among VLBW infants discharged. This mortality rate is comparable to that observed in the other neighboring countries [14, 18, 19] despite the fact that 70% of the infants we discharged weighed < 1200 g. This can be explained by the fact that a majority 59.8% of our infants were small for gestational age.

Our mortality rate was relatively high compared to that seen in the developed countries and countries that use a higher discharge weight criteria [13, 15, 16]. Studies from settings that discharged infants at much higher body weight had far lower mortality rate since those infants would be more mature with better survival mechanisms including stable body temperature while in a cot, capable to feed orally and low risk for apnoea.

The mortality among our discharge infants was inversely related to discharge weight < 1000 g (13/37), 1000–1199 (13/73) and > 1200 (6/54) this can be explained by the fact that the smaller the infant the more vulnerable they were to hypothermia, apnoea and sepsis. This observation is similar to that found in other studies that looked at discharge weight [18]. Discharging at weight < 1200 g in low resource setting may not be a safe practice.

Infants discharged at a postmenstrual age of < 35 weeks had a higher odds of dying 25/95 (OR 3.16, p 0.012) compared to those discharged at ≥35 weeks (7/69). Most studies that looked at survival after discharge in low resource settings did not look at postmenstrual age. At 35 weeks of gestation most infants have a good suck and swallowing reflex [20] and the risk of apnoea is very small [21] this explains why mortality was relatively lower among infants discharged at a postmenstrual age ≥ 35 weeks.

None of the social demographic characteristics was significantly associated with mortality in our study. This is contrary to findings from other studies that found maternal education, age, and race to be associated with outcome [16, 19]. Whereas it may be perceived that education level should improve survival, in our setting no special education on how to care for preterm babies is given to the mothers; hence, the care is same across education background. Thus it is important that mothers in low resource settings irrespective of social and economic background should be offered special training on how to handle such vulnerable babies in order to improve their chances to survive.

A majority of the infants who died did so in the first month after discharge 22/32 (68.7%). This finding is similar to that observed in other studies [14, 18, 19] and this can be explained by the fact that during the first month after discharge the mother is not yet competent in handling the infant, whereas at the same time after the first month the infant has grown and developed more survival mechanisms.

A majority of the infants who died in our study did so from suspected sepsis 16/32 (50.0%), which was not surprising. Sepsis still contributes significantly to neonatal deaths in the developing countries [4]. Whereas most studies that looked at survival of VLBW babies after discharge did not elaborate of causes of death, some suggested illness in the infants needing to attend hospital prior to death [13, 14, 19].

We also noted cot death to contribute significantly to deaths after discharge 8/32 (25%). This may be explained by the fact that majority of our infants were discharged at weight of < 1200 g and postmenstrual age of < 35 weeks. Although all our infants were discharged on oral aminophylline, the stability of the medication and whether it was actually given could not be ascertained.

Whereas it is not a common practice in low resource settings to estimate gestational age, it is important that health workers do so, since it should enable them identify babies at risk for apnoea.

## Recommendation

In order to reduce mortality among VLBW infants, they should be discharged preferably at weight ≥ 1200 g and after measures to prevent sepsis and cot death have been addressed.

### Study limitation

High loss to follow up of 13.7% and excluded infants of parents not having reliable phone contact might have skewed our findings. We could also not ascertain the exact cause of death.

### What is already known?

Preterm/VLBW infants mortality rate is high and it goes beyond the neonatal period.

### What this study adds?

Proportion of VLBW infants that died after discharge from Mulago SCBU, factors attributed to death and possible causes of death among such babies. These may be the same in other low resource settings.

## Conclusion

The mortality observed among VLBW infants discharged from Mulago Special Care Baby Unit of 19.5% is high. Factors associated with mortality include discharge weight < 1000 g, and post menstrual age at discharge of < 35 weeks. Most of the deaths were from presumed sepsis and suspected cot death.

## Abbreviations

HIV: Human immunodeficiency virus
KMC: Kangaroo mother care
SCBU: Special care baby unit
SGA: Small for gestational age
VLBW: Very low birth weight
WHO: World Health Organization
